# A new species of Fauveliopsidae (Annelida) from the North Sea

**DOI:** 10.3897/zookeys.181.2712

**Published:** 2012-04-06

**Authors:** Anna Zhadan, Margarita Atroshchenko

**Affiliations:** 1 Moscow State University, Faculty of Biology, Leninskie Gory, 1–12, Moscow, 119234 Russia; 2 State Biological Museum, Moscow, Malaya Gruzinskaya str., 15, 123242 Russia

**Keywords:** Annelida, Polychaeta, Fauveliopsidae, *Laubieriopsis norvegica*, new species, taxonomy, Norway, Northeast Atlantic

## Abstract

A new species of the genus *Laubieriopsis* Petersen, 2000 is described based on 28 specimens collected in the north-east part of the North Sea. It is characterized by fixed number of chaetigers (22), paired genital papillae, bidentate neurochaeta of chaetigers 1–4, the absence of acicular chaetae on chaetigers 5–21 and, on the last chaetiger, one acicular and three capillary chaetae enlarged and directed backward. The present study brings the number of known species of *Laubieriopsis* to five and the number of Northeast Atlantic species of this genus to two.

## Introduction

The Fauveliopsidae are a small (about 20 species) family of detritus-feeding polychaetes. They are small benthic animals, mainly found on deep bottoms down to 6000 m (Levenstein 1970), although a few species can be found above 100 m deep (Katzmann and Laubier 1974, Riser 1987, Núñez et al. 1997). Members of the family are either free-living or inhabit dead shells of scaphopods, gastropods or foraminiferans (Blake and Petersen 2000, Petersen 2000).

The family Fauveliopsidae was erected by Hartman (1971) and initially consisted of one genus, *Fauveliopsis* McIntosh, 1922; later Petersen (2000) divided this genus into two – *Fauveliopsis* and *Laubieriopsis* Petersen, 2000. Fauveliopsidae are usually grouped together with Flabelligeridae, based on the presence of a retractable anterior region of the body, but differ from Flabelligeridae by the lack of a cephalic cage, retractile oral branchiae and palps. The presence of an interramal papilla is the characteristic feature for the family, though Fauchald and Rouse (1997) considered it to be the same as the interramal papillae of Flabelligeridae and thus found no evidence for monophyly of the family. Genital papilla (single or paired) could be another characteristic feature for the family Fauveliopsidae (Petersen 2000).

The internal morphology and ultrastructure of fauveliopsids have been poorly studied. Riser (1987) described some features for *Laubieriopsis arenicola* Riser, 1987, *Laubieriopsis brevis* (Hartman, 1965)and *Fauveliopsis glabra* (Hartman, 1960). Purschke (2011) gave a detailed description of the anterior end of *Fauveliopsis* cf. *adriatica*, its brain and sensory structures. Thiel et al. (2011) described some anatomical features of *Fauveliopsis confusa* Thiel, Purschke and Böggemann, 2011. Common characters of all the studied species are a thick cuticle, a brain with four posterior lobes, longitudinal muscles forming four muscle bands, strong prostomium protractors, muscular dissepiments, two ventral mesenteries, and epidermal glands associated with parapodia.

Zhadan and Atroshchenko (2010) described the inner organization of *Laubieriopsis* sp. They examined the morphology of the body wall, introvert, and body cavities as well as the digestive, reproductive, and nervous systems. In the present work we formally describe this species.

## Material and methods

**Figure 1.**
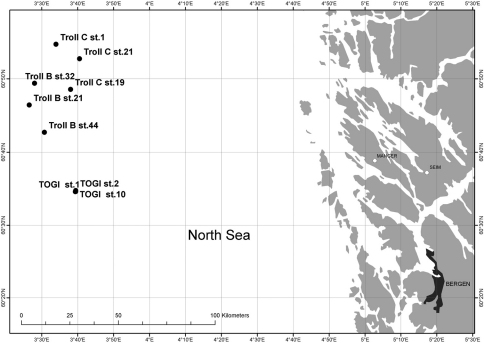
Map of stations of Akvapla-niva, 1998, showing findings of *Laubieriopsis norvegica* sp.n.

Specimens of *Laubieriopsis* sp. were collected in the North Sea (60°34'N, 03°41'E; 03°26'E, 60°54'N) (Fig. 1) by the company Akvaplan-niva in May 1998 (collector Andrey Sikorski). All specimens were fixed in a 4% formaldehyde seawater solution and later transferred to 70% ethanol. The material is stored in the Zoological Museum of M.V. Lomonosov Moscow State University (ZMMU).

Twenty-one specimens were examined under a stereomicroscope after dehydration in a glycerin series. Additional data were obtained using scanning electron microscopy: seven specimens were critical point-dried after dehydration in an ethanol series and acetone, and then coated with gold prior to examination with a Scanning Electron Microscope HITACHI S-405 A and Camscan-S2.

## Results

### Genus Laubieriopsis

Petersen, 2000

http://species-id.net/wiki/Laubieriopsis

#### Diagnosis.

Body cylindrical, weakly divided into two regions by segment shape and type of chaetae. Fixed number of segments. Cuticle smooth, without papillae or papillae minute and inconspicuous. Chaetae include capillaries and acicular types, some might be bidentate. Interramal papillae small, mostly sessile. Last segment similar in size to preceding ones, often bilobed, pygidium retracted inside it.

### Laubieriopsis norvegica sp. n.

urn:lsid:zoobank.org:act:5D46C0C3-6974-4683-81BF-C09964602392

http://species-id.net/wiki/Laubieriopsis_norvegica

Figs 2 3 4

Laubieriopsis sp. Zhadan and Atroshchenko 2010: 876-885, Figs 1–4

#### Type locality.

Norway, North Sea, 60°52.69'N, 03°40.48'E, 340 m, mud. Akvaplan-niva, Troll C stn. 21–4; 14.05.1998. coll. A. Sikorski.

#### Type material.

Holotype female with 32 oocytes, 6.8 mm long; width in dorsal view on chaetiger 4–400 µm, stored in 70% alcohol. Original label: “Spec.: Polychaeta indet., stn.: 21–4, Date: 14.05. Troll C 1998. Akvaplan-niva A.Sikorski”. ZMMU № W141 HOLOTYPE, *Laubieriopsis norvegica* (printed label).

Paratypes: Akvaplan-niva Troll C stn. 21–4, 60°52.69'N, 03°40.48'E, 14.05.1998, 340 m, mud, one specimen without visible gametes, ZMMU № W142. Akvaplan-niva Troll C stn. 19–3, 60°48.54'N, 03°38.03'E, 14.05.1998, 334 m, three specimens, two without visible gametes, one female with 39 oocytes, ZMMU № W143. Akvaplan-niva Troll C stn 1–5, 60°54.65'N, 03°33.95'E, 15.05.1998, 341 m, two specimens without visible gametes, ZMMU № W144. Akvaplan-niva TOGI stn.10–4, 60°34.58'N, 03°39.34'E, 10.05.1998, 304 m, four specimens, three without visible gametes, one male with sperm, ZMMU № W144. Akvaplan-niva TOGI stn.1–4, 60°34.59'N, 03°39.45'E, 9.05.1998, 305 m, three specimens with small oocytes, ZMMU № W146. Akvaplan-niva TOGI stn. 2–4, 60°34.72'N, 03°39.46' E, 9.05.1998, 305 m, one specimen, male with sperm, ZMMU № W147. Akvaplan-niva Troll B, stn.32–3, 60°49.37'N, 03°27.98'E, 14.05.1998, 335 m, three specimens, two females with oocytes, one without visible gametes, ZMMU № W148. Akvaplan-niva Troll B, stn. 21–3, 60°46.40'N, 03°26.51'E, 14.05.1998, 322 m, two specimens without visible gametes, ZMMU № W149. Akvaplan-niva Troll B, stn.44–4, 60°42.68'N, 03°30.74'E, 14.05.1998, 319 m, one specimen without visible gametes, ZMMU № W150.

#### Diagnosis.

Adult specimens with 22 chaetigers. First four chaetigers with sigmoidal acicular chaetae, neurochaetae often bidentate. Chaetigers 5–21 without acicular chaetae, with one thin capillary chaeta and one very small and thin additional chaeta in each ramus. Chaetiger 22 with one thick acicular notochaetae and three capillary neurochaetae. Two weltlike genital papillae present at posterior edge of segment 8.

#### Description.

**Figure 2.**
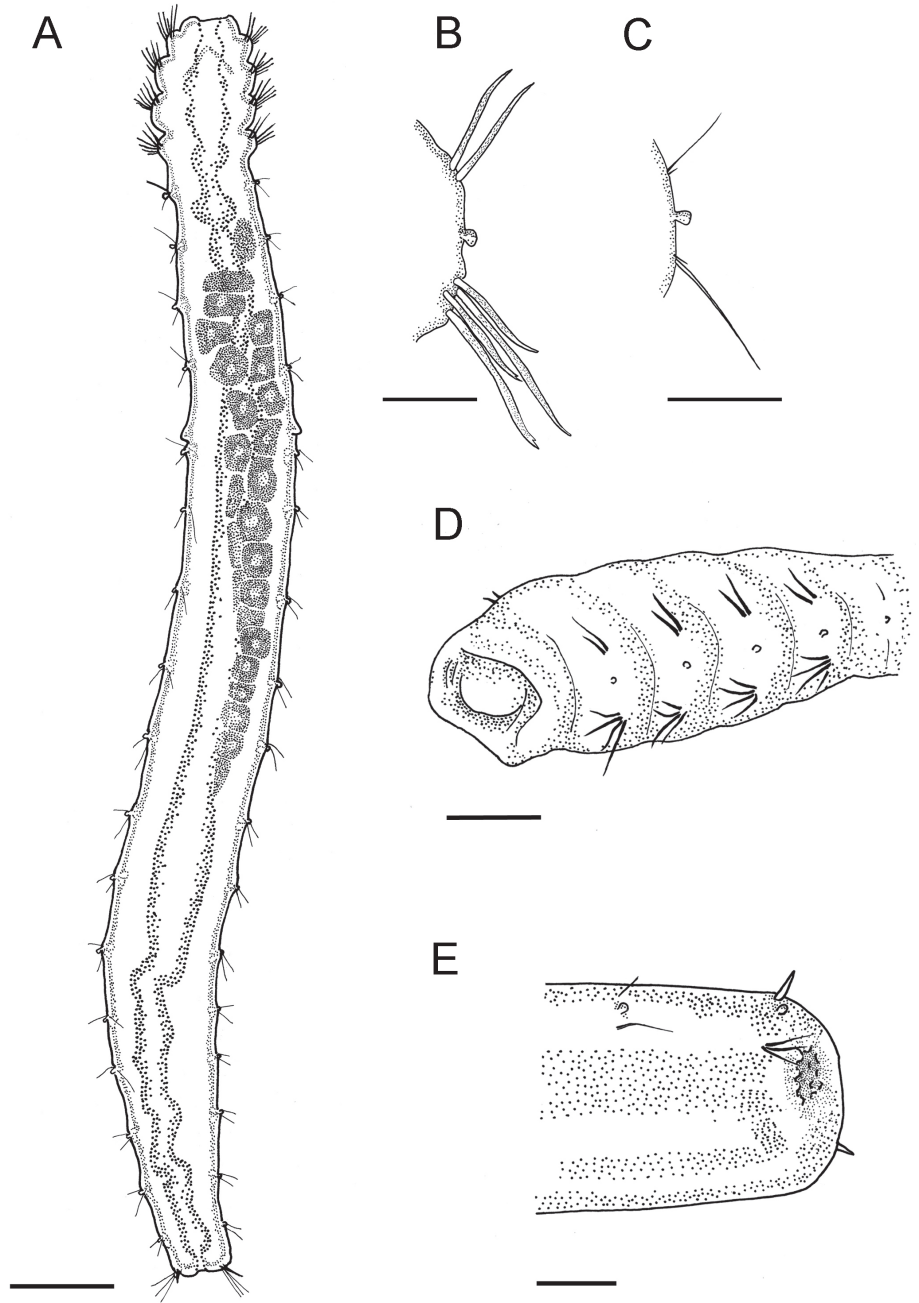
*Laubieriopsis norvegica* sp.n. **A** dorsal view of female with oocytes **B** parapodia of chaetiger 3 **C** parapodia of chaetiger 14 **D** anterolateral view of anterior end (prostomium visible) **E** dorsolateral view of posterior end. Scale (μm): **A** – 400, **B, D, E** – 100, **C** – 30.

**Figure 3.**
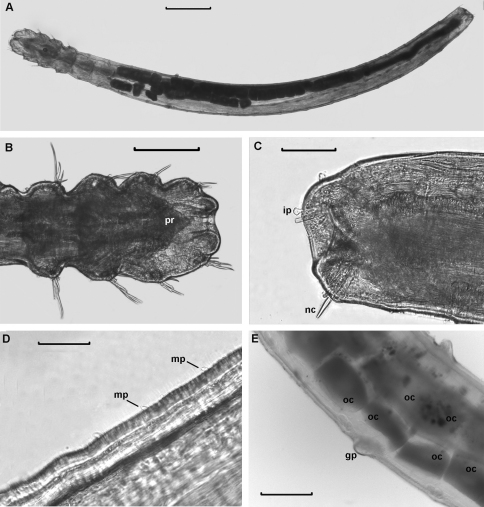
*Laubieriopsis norvegica* sp.n., **A** entire view from the dorsal side **B** anterior end, ventral view **C** posterior end, ventral view **D** micropapillae **E** area of chaetigers 7–9, genital papillae. Scale (μm): **A** – 500; **B**– 200; **C, E** – 100; **D**– 50.

Adult specimens 6–8 mm long and 0.35–0.4 mm wide with 22 chaetigers. Only one specimen has 23 chaetigers. Body slender, often c–shaped or s-shaped by fixation, colorless in preserved material. Four anteriormost chaetigers swollen, following segments cylindrical, without clear borders (Fig. 2A, 3A, B).

**Figure 4.**
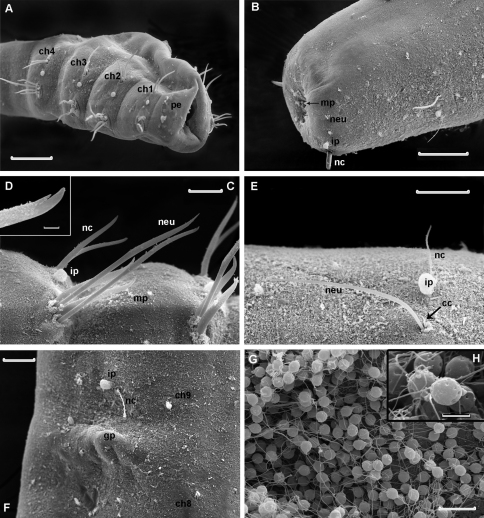
*Laubieriopsis norvegica* sp.n. **A** anterior end, lateral view **B** posterior end, ventro-lateral view **C** parapodia of chaetigers 2–3 **D** tip of neurochaeta enlarged **E** parapodia of chaetiger 12 **F** genital papilla and parapodia of chaetiger 9; neurochaetae are broken **G, H** sperm cell. Scale (μm): **A, B** – 100, **C**–30, **D** – 3, **E, F** – 30, **G** – 10, **H** – 2. Abbreviation: **cc** accessory chaeta, **ch1–ch4** chaetigers 1–4, **ch8** chaetiger 8, **ch9** chaetiger 9, **gp** genital papilla, **ip** interramal papilla, **mp** micropapilla, **neu** neurochaeta, **nc** notochaeta, **oc** oocyte, **pe** peristomium, **pr** prostomium

Cuticle thick (about 8 μm), smooth, with very thin ring wrinkles, bearing scattered minute inconspicuous micropapillae (Fig. 3D, 4C), visible under higher magnification and in SEM. Relatively larger micropapillae surrounding inverted parts – prostomium and pygidium (Fig. 4B).

Ventral nerve cord visible by transparency. Ganglia longitudinally elongated and indistinctly separated from each other.

Prostomium small, from round to triangular, lacking appendages, with ciliary nuchal organs, without eyes (Fig. 2D, 3B). In most specimens prostomium completely retracted into peristomium (Fig. 4A). Peristomium forms complete ring; it bears micropapillae.

Parapodia biramous, best developed on chaetigers 1–4 and hardly distinct on posteriormost segments except last one (22). Interramal papillae short–stalked, pyriform, situated midway between noto- and neuropodia (Fig. 3C, 4A, B, C, E, F). Two epidermal glands visible in each parapodium.

Chaetigers 1–4 bear thick sigmoidal hirsute chaetae. Two chaetae in notopodia and four (2 long and 2 shorter ones) in neuropodia (Fig. 2B, 4C). Some specimens possess five instead of four neurochaetae in anterior segments. Neurochaetae mostly bidentate (Fig. 4D), notochaetae usually unidentate, sometimes slightly bidentate. Some specimens possess 3–4 bidentate neurochaetae whereas others have only 1–2 bidentate neurochaetae. Both short and long neurochaetae can be bidentate or unidentate. Two specimens have transitional fifth segment with chaetae more similar to acicular chaetae of anterior four segments than to thin capillary chaetae of rest of the body.

Chaetigers 5–21 bear one thin capillary chaeta and one accessory very short, thin and barely distinct capillary chaeta per ramus (Fig. 2C, 4E, F). Notopodial chaetae thinner and shorter than neuropodial ones; accessory chaetae in notopodia often absent.

Chaetiger 22 bears one thick acicular chaeta in notopodia and three capillary chaetae in neuropodia (Fig. 2E, 3C, 4B).

Paired genital papillae (Fig. 3E, 4F) situated at posterior edge of chaetiger 8 in all studied individuals with gametes as well as in specimens without recognizable gametes, except for one specimen with unpaired genital papilla.

Pygidium retracted within last chaetiger, anus terminal. Boundary between distal part of last segment and pygidium indistinct. Inverted part (distal to chaetae) with a number of conical papillae, which are larger than in rest of body (Fig. 2E, 3C, 4B).

Oocytes up to 90 μm in diameter, arranged in two ovisacs, extend through chaetigers 6–15, each contains 20–40 oocytes (Fig. 2A, 3A, E).

Sperm cells hardly visible through body wall, but observed in SEM in body cavity of dissected specimen. They have rounded heads about 3 μm in diameter, an acrosome with elongated distal part and free flagellum at least 10 μm long (Fig. 4G, H).

#### Etymology.

The species name refers to its type locality.

#### Distribution.

Northeast part of the North Sea.

#### Ecology.

Inhabits muddy sediments with small admixture (0.8 – 7.7 %) of fine and medium sand, in depth of 300–350 m.

#### Discussion.

*Laubieriopsis norvegica* sp. n. differs from all previously described species of the family Fauveliopsidae by the highly reduced chaetal bunds in median and posterior parapodia. Whereas all fauveliopsid species have one, rarely two acicular spines in each parapodial ramus, noto- and neuropodia of chaetigers 5–21 in *Laubieriopsis norvegica* sp. n bear one fine, slender capillary and one even smaller chaeta which may be absent.

Species of the genus *Laubieriopsis* are characterized by a fixed number of chaetigers; previously described species have 16, 21 and 25 chaetigers (Petersen 2000). *Laubieriopsis norvegica* sp.n. is most similar to *Laubieriopsis cabiochi* (Amoureux, 1982) in the number of chaetigers (22 and 21, respectively) but it has significant differences. *Laubieriopsis norvegica* have paired genital papillae while in *Laubieriopsis cabiochi* the genital papilla is unpaired. Paired genital papillae were observed in some other species of the genus, for example *Laubieriopsis arenicola*. Parapodia of the last chaetiger in *Laubieriopsis norvegica* differ from the other parapodia, bearing one thick acicular chaeta and three capillary chaetae. Enlarged and backward-directed chaetae of the terminal chaetiger – “pygidial cage” – were described also for other *Laubieriopsis* species (*Laubieriopsis brevis*, *Laubieriopsis arenicola*) (Riser 1987, Petersen 2000). *Laubieriopsis arenicola* differs from *Laubieriopsis norvegica* by having 25 thoracic chaetigers and in the location of genital papillae on chaetiger 7.

Our study confirms Petersen’s (2000) statement on characters useful for distinguishing species in the genus *Laubieriopsis*: number of chaetigers, number of anterior chaetigers with acicular chaetae, presence of bidentate anterior neurochaetae, enlarged and directed backward chaetae of the last chaetiger, and paired/unpaired genital papilla. Interestingly, one of 28 specimens studied here has 23 instead 22 thoracic chaetigers and another one has an unpaired genital papilla. As intraspecific variability was noted for all characters (present work, Petersen 2000), description of new as well as redescription of existing taxa should be based on examination of several specimens and the whole complex of characters.

There was only one species of the genus *Laubieriopsis* previously known from the Northeast Atlantic, *Laubieriopsis cabiochi*. The present study increases the number of known species of *Laubieriopsis* to five and the number of Northeast Atlantic species of this genus to two.

## Supplementary Material

XML Treatment for Laubieriopsis

XML Treatment for Laubieriopsis norvegica
